# Sca-1+ Cardiac Stem Cells Mediate Acute Cardioprotection via Paracrine Factor SDF-1 following Myocardial Ischemia/Reperfusion

**DOI:** 10.1371/journal.pone.0029246

**Published:** 2011-12-15

**Authors:** Chunyan Huang, Hongmei Gu, Qing Yu, Mariuxi C. Manukyan, Jeffrey A. Poynter, Meijing Wang

**Affiliations:** 1 Department of Surgery, Indiana University School of Medicine, Indianapolis, Indiana, United States of America; 2 Department of Pediatrics, Indiana University School of Medicine, Indianapolis, Indiana, United States of America; 3 Herman B. Wells Center for Pediatric Research, Indiana University School of Medicine, Indianapolis, Indiana, United States of America; Leiden University Medical Center, The Netherlands

## Abstract

**Background:**

Cardiac stem cells (CSCs) promote myocardial recovery following ischemia through their regenerative properties. However, little is known regarding the implication of paracrine action by CSCs in the setting of myocardial ischemia/reperfusion (I/R) injury although it is well documented that non-cardiac stem cells mediate cardioprotection via the production of paracrine protective factors. Here, we studied whether CSCs could initiate acute protection following global myocardial I/R via paracrine effect and what component from CSCs is critical to this protection.

**Methodology/Principal Findings:**

A murine model of global myocardial I/R was utilized to investigate paracrine effect of Sca-1+ CSCs on cardiac function. Intracoronary delivery of CSCs or CSC conditioned medium (CSC CM) prior to ischemia significantly improved myocardial function following I/R. siRNA targeting of VEGF in CSCs did not affect CSC-preserved myocardial function in response to I/R injury. However, differentiation of CSCs to cardiomyocytes (DCSCs) abolished this protection. Through direct comparison of the protein expression profiles of CSCs and DCSCs, SDF-1 was identified as one of the dominant paracrine factors secreted by CSCs. Blockade of the SDF-1 receptor by AMD3100 or downregulated SDF-1 expression in CSCs by specific SDF-1 siRNA dramatically impaired CSC-induced improvement in cardiac function and increased myocardial damage following I/R. Of note, CSC treatment increased myocardial STAT3 activation after I/R, whereas downregulation of SDF-1 action by blockade of the SDF-1 receptor or SDF-1 siRNA transfection abolished CSC-induced STAT3 activation. In addition, inhibition of STAT3 activation attenuated CSC-mediated cardioprotection following I/R. Finally, post-ischemic infusion of CSC CM was shown to significantly protect I/R-caused myocardial dysfunction.

**Conclusions/Significance:**

This study suggests that CSCs acutely improve post-ischemic myocardial function through paracrine factor SDF-1 and up-regulated myocardial STAT3 activation.

## Introduction

Despite advances in the treatment of myocardial infarction (MI), ischemic heart disease remains a leading cause of death world-wide. MI causes an irreversible loss of myocardium followed by the replacement of scar tissue. Stem cell-based therapy, therefore, holds tremendous promise in the treatment of myocardial ischemia due to their regeneration potential. However, accumulated evidence has demonstrated that cardiac and vascular differentiation of implanted cells plays a less important role in mediating cardioprotection than previously thought. More and more studies have pointed out that stem cells mediate their beneficial effects mainly through production of paracrine factors [1]–[5]. Stem cell-secreted protective molecules have been reported to increase cell survival, reduce inflammation, promote local angiogenesis and improve myocardial function after MI [1]–[4]. Studies from our group have also demonstrated that infusion of mesenchymal stem cells (MSCs) into isolated rodent hearts prior to ischemia improved myocardial function after 25-minute ischemia followed by 40-minutes reperfusion, clearly indicating that MSCs acutely protect the heart without cell differentiation [5]–[7].

On the other hand, compelling evidence has suggested that the adult heart contains cardiac stem cells (CSCs), which have been isolated in many species based on stem cell markers such as Sca-1, c-Kit, Isl-1, and/or MDR1 [8]–[17]. Prior research has primarily focused on investigating the differentiation capacities of CSCs and their ability to differentiate into cardiomyocytes, smooth muscle cells and endothelial cells after MI [8], [12], [14], [15]. However, little information exists regarding the paracrine action of CSCs in cardioprotection following myocardial ischemia/reperfusion (I/R) injury. In fact, CSCs are capable of producing substantial quantities of paracrine molecules [18]–[20]. Therefore, understanding CSC-derived paracrine action is of value to promote protective effect following myocardial ischemia. To date, although a majority of studies have shown that stem cell-secreted growth factors VEGF, HGF and IGF-1 are able to protect the myocardium against ischemic injury, it is largely unknown what component derived from CSCs contributes to the improvement of cardiac function in response to myocardial I/R injury.

Therefore, to determine the role of CSC-derived paracrine action in modulating acute cardioprotection following I/R, we utilized an isolated heart perfusion system (Langendorff) which excludes the effects of other blood-borne elements on the actions of CSCs and avoids the potential confounding effects of systemic actions when the heart is subjected to I/R in vivo. In the present study, we reported that intracoronary delivery of in-vitro expanded Sca-1+ CSCs into isolated mouse hearts prior to ischemia improved myocardial function following I/R injury. In addition, the stromal-cell derived factor-1α (SDF-1) was identified as one of the dominant paracrine factors secreted by CSCs. Furthermore, we demonstrated that SDF-1 played a critical role in CSC-mediated acute cardioprotection through the signal transducer and activator of transcription 3 (STAT3) signaling.

## Results

### Cardiac stem cell characteristics

**Figure 1:**
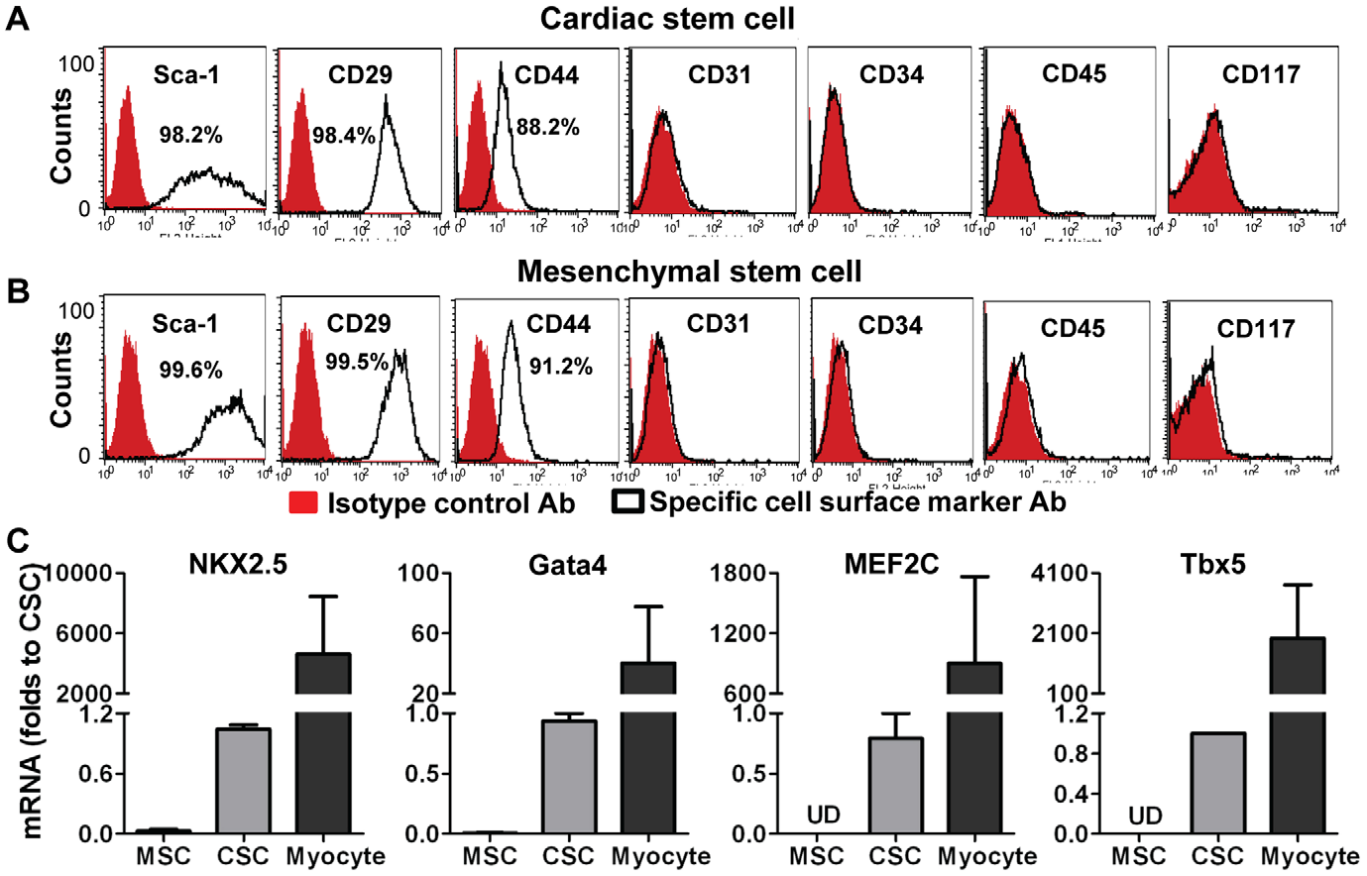
Identification of cardiac stem cell (CSC) characteristics. A, Flow cytometry assay indicated expression of cell surface markers in CSCs. B, Expression of cell surface markers in mesenchymal stem cells (MSCs). C, RT Real-time PCR data showed mRNA levels of cardiac specific transcription factors in CSCs, MSCs and cardiomyocytes (isolated from adult mouse heart as a positive control). Mean ± SEM, n = 3 individual experiments. UD: Undetectable.

In order to identify whether the cells we isolated from murine hearts had cardiac stem cell characteristics, we first determined the expression of stem cell markers. Flow cytometric data revealed that these cells were positive for Sca-1 (>98%), CD29 (>98%) and CD44 (∼90%), but negative for hematopoietic stem cell markers CD34, CD45 and the endothelial marker CD31, as well as CD117 (Fig. 1A). CSCs expressed similar levels of stem cell markers as MSCs did (Fig. 1B). However, RT-qPCR analysis indicated that CSCs exhibited much higher transcription levels of the cardiac specific transcription factors NKX2.5, Gata4, MEF2c and Tbx5 compared to MSCs (Fig. 1C).

### CSCs improved myocardial function through paracrine action following I/R

**Figure 2:**
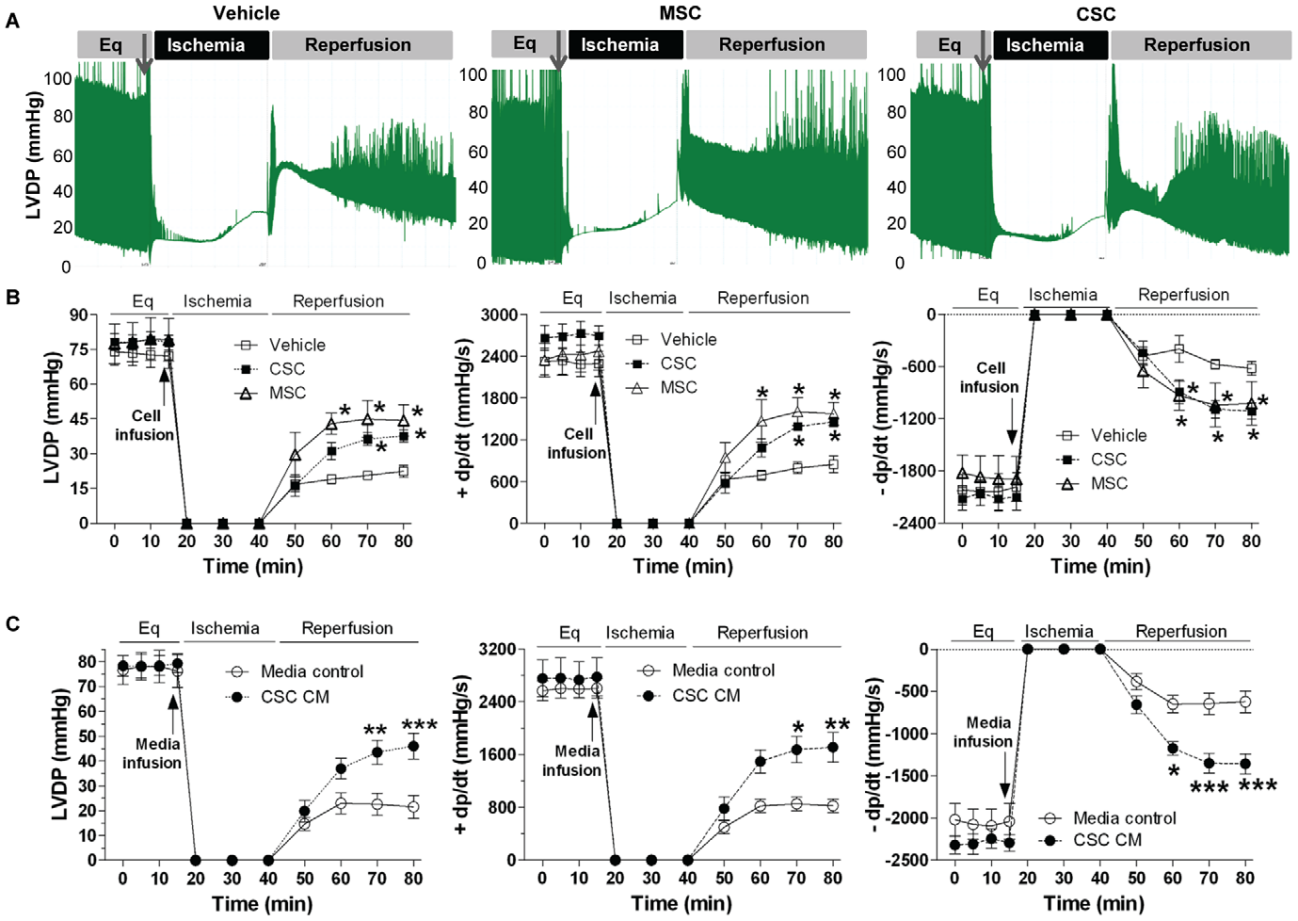
CSC-derived paracrine protection in myocardial function following I/R. Left ventricular developed pressure (LVDP) recording trace was shown during equilibration (Eq), global ischemia and reperfusion (A). Changes of LVDP and +/− dP/dt following I/R in groups of CSC, MSC and vehicle (B), and groups of CSC CM and media control (C). Mean ± SEM, n = 5–6/group, *p<0.05, **p<0.01, ***p<0.001 vs. vehicle or media controls. CM: conditioned medium.

Studies from our group and others have demonstrated that implanted MSCs improved myocardial function after ischemic injury through the release of protective factors [1], [2], [5], [6]. To investigate the paracrine effect of CSCs on cardiac function, we infused CSCs (0.1×10^6^ in 1 ml of perfusate) into isolated mouse hearts prior to ischemia through intracoronary delivery. Expectedly, infusion of MSCs acutely protected post-ischemic myocardial function, which was in agreement with our previous observations [5]–[7]. Importantly, significantly improved myocardial function of LVDP and +/− dP/dt was noticed in the CSC-pretreated group compared to vehicle after I/R (Fig. 2A, B), suggesting that CSCs are able to mediate acute cardioprotection following I/R like MSCs did. To verify that CSC-derived paracrine factors are responsible for this acute protection, we collected conditioned medium (CM) from CSC culture and injected them into isolated hearts. Similarly, infusion of CSC CM prior to ischemia improved post-ischemic myocardial function of LVDP and +/− dP/dt compared to media control (Fig. 2C), implying that some protective factors are secreted from CSCs.

### VEGF was not critical to CSC-mediated acute protection following myocardial I/R injury

**Figure 3:**
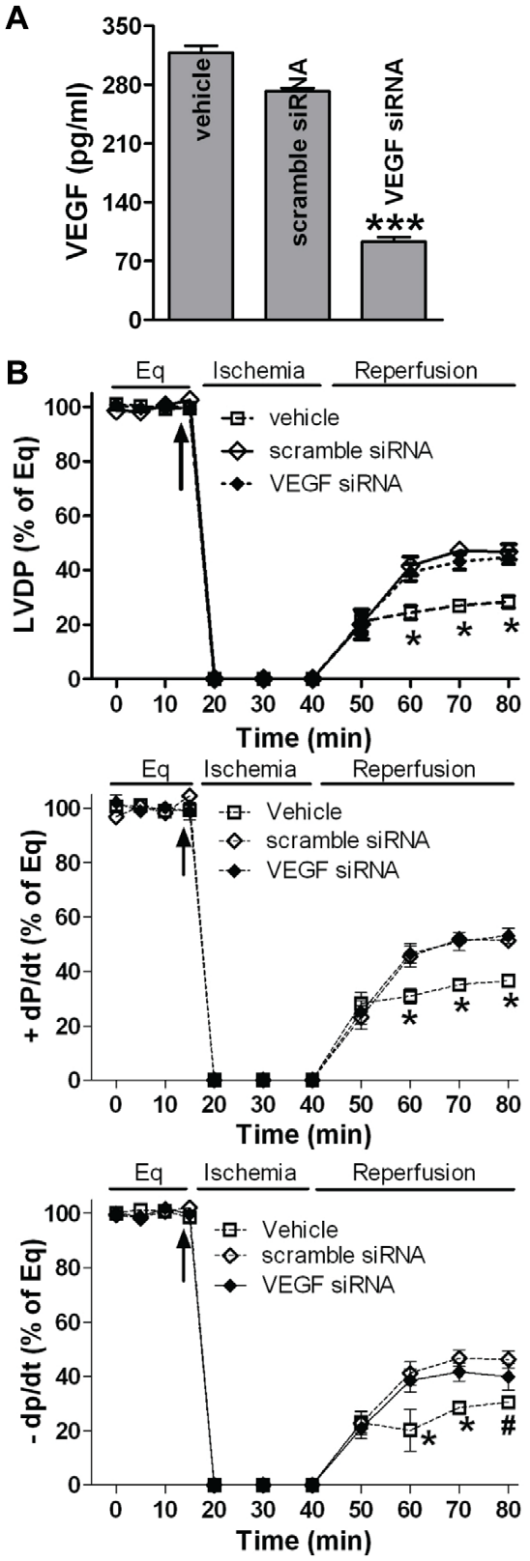
Role of VEGF in CSC-mediated acute protection following myocardial I/R injury. A, Production of VEGF was determined in siRNA transfected CSCs by ELISA. Mean ± SEM, N = 3, ***p<0.001 vs. vehicle or scramble siRNA. B, VEGF siRNA transfection did not attenuate CSC-improved myocardial function following I/R. Mean ± SEM, N = 5–6/group, *p<0.01 vs. scramble siRNA and VEGF siRNA, #p<0.05 vs. scramble siRNA only.

Next, we determined which molecule(s) from CSCs is responsible for this acute protection following myocardial I/R injury. Given that VEGF plays a critical role in stem cell-mediated paracrine protection [7], we hypothesized that VEGF derived from CSCs might play a role in CSC-mediated acute protection following I/R injury. To verify this, siRNAs of VEGF and scramble control were transfected into CSCs. After 2 days, VEGF production in the transfected CSCs was measured. Interestingly, although VEGF siRNA reduced CSC production of VEGF (Fig. 3A), these transfected CSCs were still able to protect post-ischemic myocardial function as scramble siRNA-transfected CSCs did (Fig. 3B), indicating that CSC-derived VEGF may not be critical to this acute protection.

### Differentiated CSCs (DSCSs) did not protect cardiac function in response to I/R

**Figure 4:**
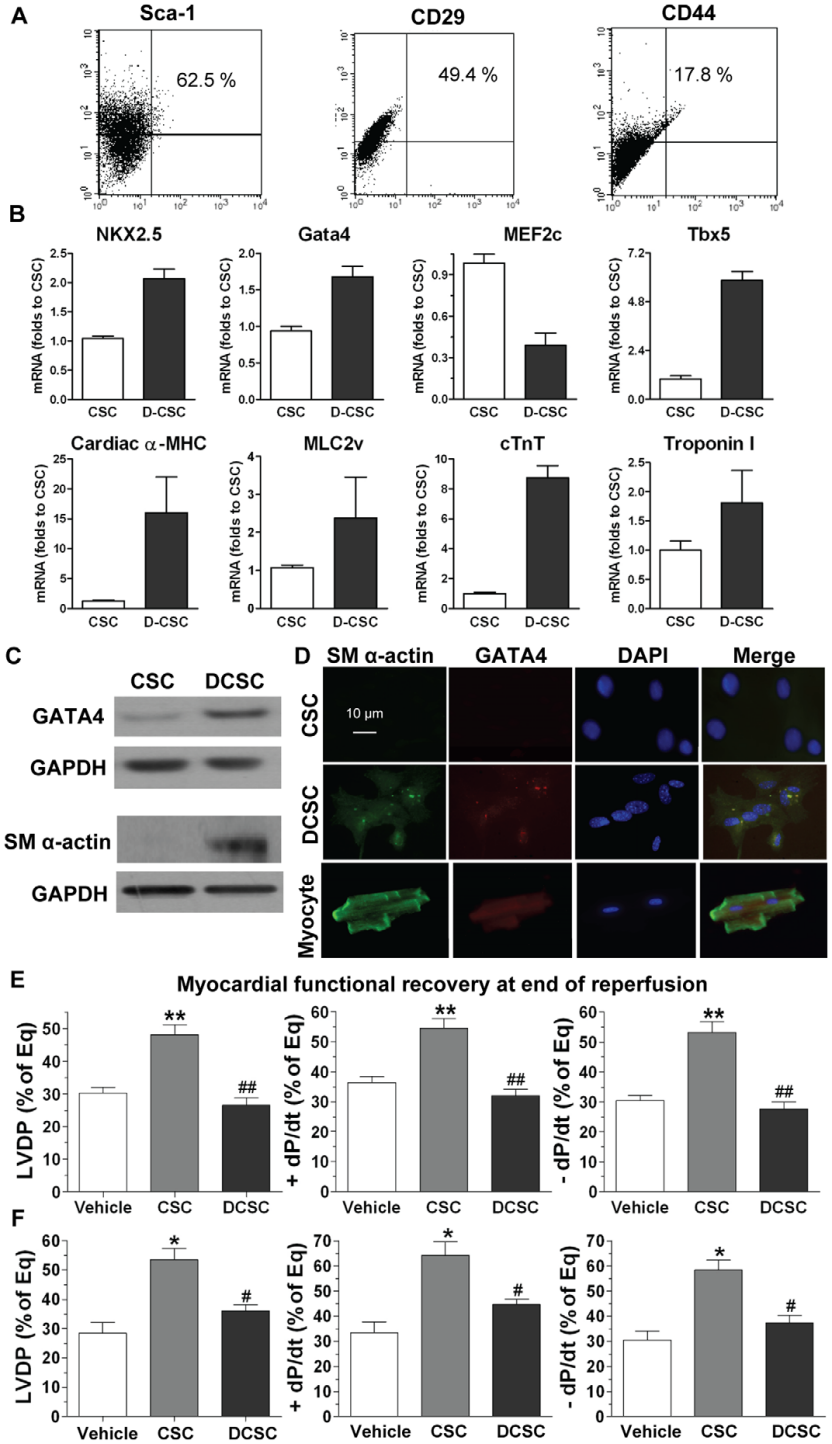
Role of differentiated CSCs (DCSCs) in myocardial functional recovery following I/R. A, Expression of Sca-1, CD29 and CD44 in DCSCs was determined by Flow cytometry assay. B, RT Real-time PCR analysis indicated transcription levels of cardiac specific transcription factors and genes in DCSCs. C, Western blot assay demonstrated increased expression of Gata4 and sarcomeric (SM) α-actin in DCSCs compared to un-induced CSCs. D, Expression of SM α-actin (green) and Gata4 (red) was observed in DCSCs by Immunofluoresence assay (Magnification 400X). Nucleus was stained with DAPI (blue). Myocardial functional recovery at end of reperfusion was represented as % of equilibration (Eq) in groups treated with vehicle, CSC and DCSC (E) or media control, CSC CM and DCSC CM (F). Mean ± SEM, n = 5–6/group, *p<0.01, **p<0.001 vs. Vehicle or Media C, #p<0.01, ##p<0.001 vs. CSC or CSC CM. CM: conditioned medium.

To further identify what component plays an important role in CSC-mediated paracrine protection, we determined whether DCSCs still possessed this acute protective effect. DCSCs were obtained by culturing CSCs in cardiomyocyte differentiation medium for 9–10 days. DCSCs exhibited decreased expression of Sca-1, CD29 and CD44 by Flow cytometric assay (Fig. 4A). In addition, higher mRNA levels of NKX2.5, Gata4 and Tbx5, as well as cardiac α-MHC, MLC2v, cTnT and Troponin I were observed in DCSCs compared to un-induced CSCs (Fig. 4B). Furthermore, DCSCs showed increased protein levels of Gata4 and sarcomeric (SM) α-actin by Western blot analysis and Immunofluorescent assay (Fig. 4C, D).

After identified their properties, the same amount of DCSCs (0.1×10^6^ in 1 ml of perfusate) was injected into isolated mouse hearts before ischemia. Interestingly, pretreatment with DCSCs did not improve myocardial function following I/R as CSCs did. Markedly decreased post-ischemic LVDP and +/− dP/dt were observed in the DCSC-treated group compared to CSC group (Fig. 4E). Given size of cardiomyocytes being much larger than that of stem cells [11], [21], [22], the differentiation process may result in an enlarged size of DCSCs. Therefore, to obviate possible coronary embolism by DCSC infusion that may limit blood flow, exacerbating I/R-induced myocardial damage and consequent deterioration of cardiac function, we further utilized DCSC CM. Consistent with the result from infusion of DCSCs, pretreatment with DCSC CM did not protect post-ischemic cardiac function as CSC CM did (Fig. 4F). These data suggest that protective factors are secreted from CSCs, but not from DCSCs.

### SDF-1 was a dominant paracrine factor in CSCs

**Figure 5:**
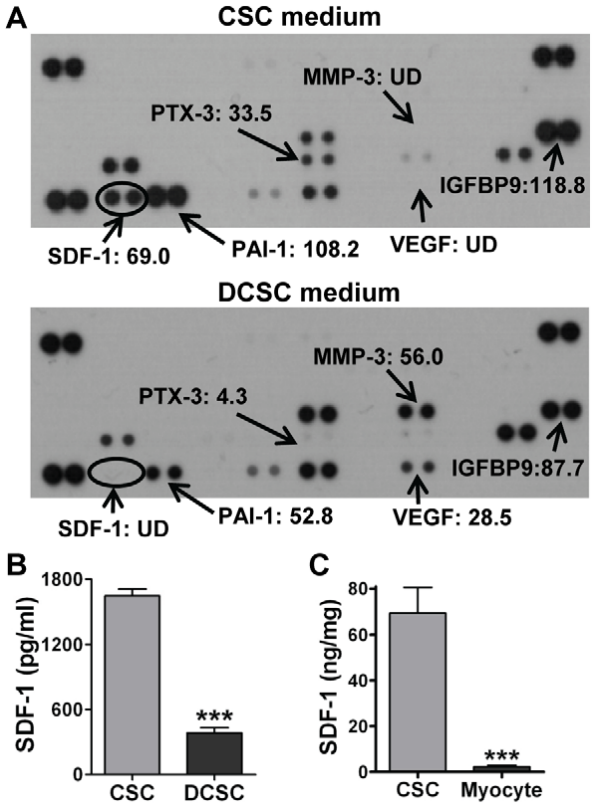
Determination of dominant paracrine factor(s) in CSCs. A, Cytokine antibody array was performed in conditioned medium (CM) from CSCs and DCSCs. Each number represented the fold increase of cytokine expression compared to the negative control (medium). B, CSC- and DCSC-secreted SDF-1 was determined in supernatants by ELISA. C, The SDF-1 expression in CSCs and cardiomyocytes was analyzed in cell lysates using ELISA. Mean ± SEM, N = 3–6/groups, ***p<0.001 vs. CSC. UD: Undetectable.

**Figure 6:**
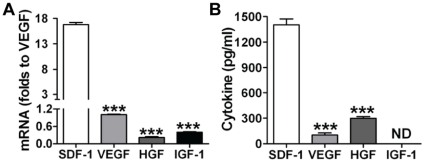
CSCs expressed much higher mRNA (A) and protein levels (B) of SDF-1 compared to production of VEGF, HGF and IGF-1. Mean ± SEM, N = 4 individual experiments, ***p<0.001 vs. SDF-1.

To determine what protective paracrine factors are different between CSCs and DCSCs, a cytokine antibody array was performed on the cell culture media. Different patterns of paracrine factor production were observed between CSCs and DCSCs (Fig. 5A). Much higher levels of SDF-1, PAI-1, PTX-3 and IGFBP-9 were found in CSC CM, whereas greater production of MMP-3 and VEGF existed in DCSC CM. Among these, SDF-1 was the most abundant factor in CSC CM compared to DCSC CM. We further confirmed that CSCs produced higher levels of SDF-1 compared to DCSCs using ELISA (Fig. 5B). In addition, CSCs expressed a 30-fold higher level of SDF-1 compared to cardiomyocytes (CSC 69.3±11.2 ng/mg vs. Myocyte 2.3±0.6 ng/mg) (Fig. 5C), suggesting that CSC differentiated to cardiomyocytes would decrease SDF-1 production. Furthermore, with respect to the capacity of producing other well-studied paracrine molecules, CSCs exhibited much lower mRNA and protein levels of VEGF, HGF and IGF-1 than their SDF-1 expression (Fig. 6A, B). These results together indicated that SDF-1 was a relatively abundant factor in CSCs.

### Role of CSC-derived SDF-1 in acute protection following I/R

**Figure 7:**
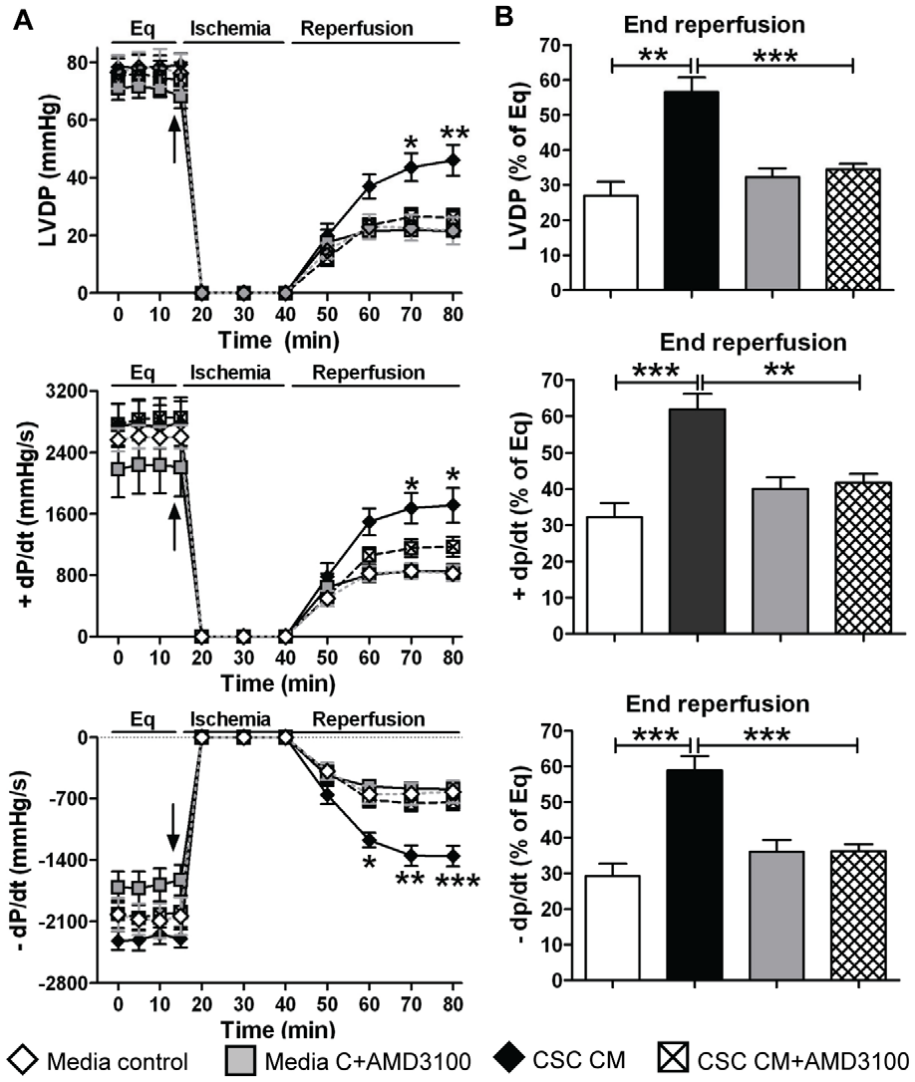
CSC-derived SDF-1 in mediating acute protection following I/R. A, Changes of LVDP and +/− dP/dt following I/R in groups of media control and CSC CM with or without AMD3100, an inhibitor of the SDF-1 receptor. B, Myocardial functional recovery at end of reperfusion was shown as % of Eq (equilibration). Mean ± SEM, n = 5–6/group, *p<0.05, **p<0.01, ***p<0.001. CM: conditioned medium.

**Figure 8:**
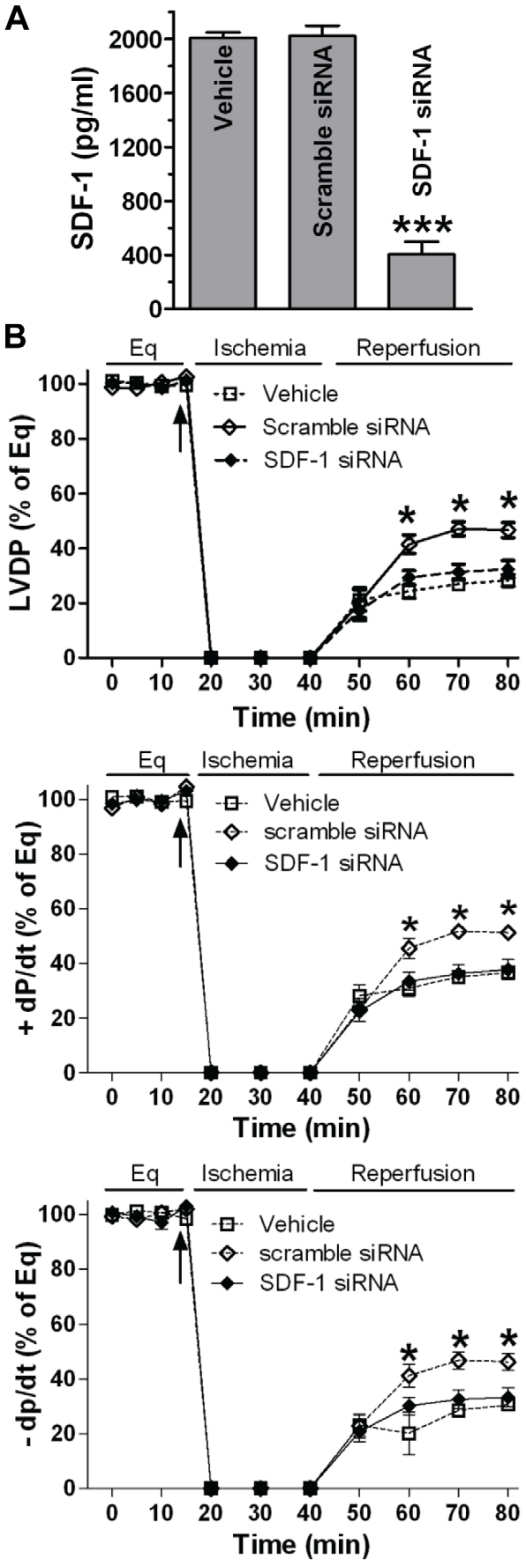
Decreased SDF-1 in CSC-mediated cardioprotection following acute I/R injury. A, Production of SDF-1 was determined in siRNA transfected CSCs by ELISA. Mean ± SEM, N = 4, ***p<0.001 vs. Vehicle and Scramble siRNA. B, Changes of LVDP and +/− dP/dt following I/R in groups of vehicle and CSCs transfected with specific siRNAs. Mean ± SEM, N = 5–6/group, *p<0.01 vs. Vehicle or SDF-1 siRNA.

We next investigated the role of CSC-derived SDF-1 in modulating cardioprotection. SDF-1 has been shown to exert its biological function through binding to its cognate receptor, CXCR4, which is present in cardiac tissue [23]. Our recent studies also confirmed that CXCR4 was expressed in isolated rodent hearts [24], [25]. Blockade of the SDF-1 receptor by AMD3100 dramatically impaired CSC-mediated protection of myocardial function to the levels seen in vehicle group and significantly decreased post-ischemic recovery of LVDP and +/− dP/dt compared to CSC CM group (Fig. 7). To verify this result, we utilized SDF-1 siRNA-transfected CSCs. SDF-1 siRNA decreased CSC production of SDF-1 by more than 80%, whereas scramble siRNA did not affect CSC-secreted SDF-1(Fig. 8A). Consistent with the result of AMD3100, a decrease in CSC production of SDF-1 abolished CSC-protected myocardial function following I/R, whereas scramble siRNA did not attenuate CSC-improved myocardial function (Fig. 8B). These data suggest that SDF-1 is a critical factor in CSC-mediated acute protection of myocardial function.

**Figure 9:**
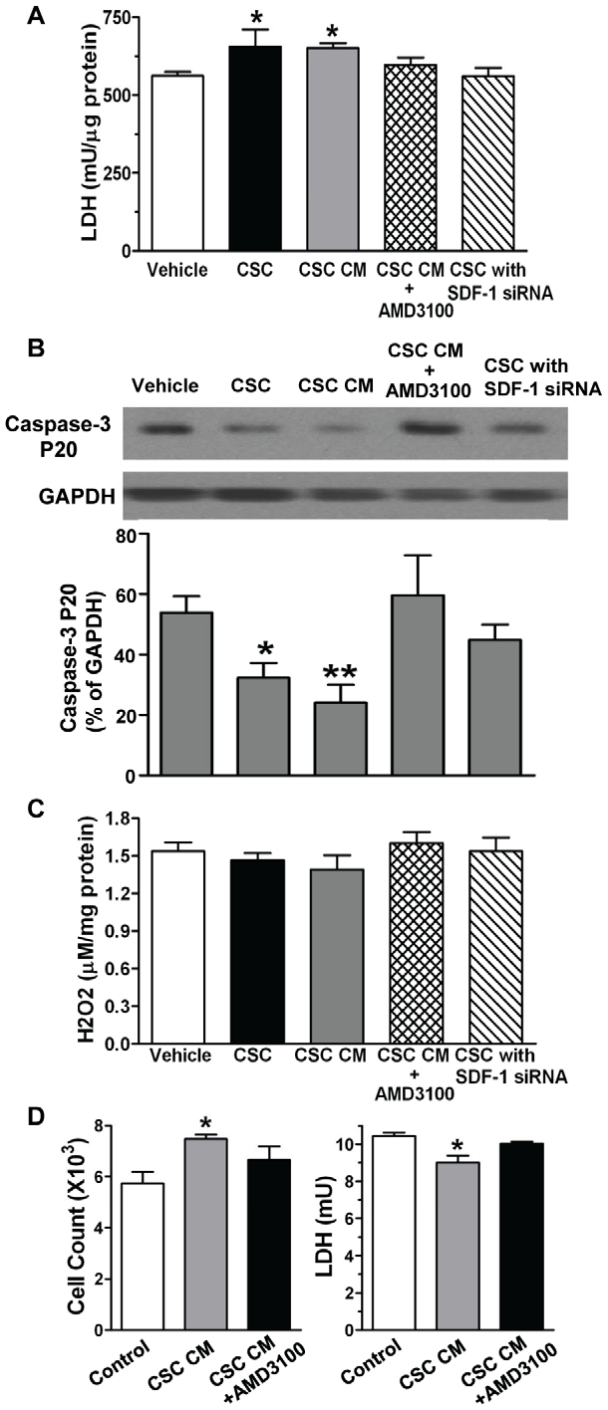
CSC-derived SDF-1 in the attenuation of cellular injury. A, Remaining LDH levels in cardiac tissue were determined after I/R. B, Western blot analysis indicated cleaved caspase-3 levels after I/R. Shown is representative immunoblots in each groups (one lane/group). Bar graph represents relative levels of Caspase-3 P20 (% of GAPDH). C, Myocardial hydrogen peroxide (H2O2) production was analyzed after I/R. A–C: Mean ± SEM, N = 4–5/group, *p<0.05, **p<0.01 vs. Vehicle. D, Cardiomyocyte (H9c2) viability and LDH levels in supernatant were determined after 24-hr of hypoxia in groups of vehicle and CSC CM with or without AMD3100. Mean ± SEM, N = 3, *p<0.05 vs. vehicle.

To further study the role of CSC-derived SDF-1 in alleviation of myocardial damage, we assessed lactate dehydrogenase (LDH) levels in cardiac tissue after I/R. Greater myocardial damage from I/R leads to higher levels of LDH in the coronary effluent and lower remaining LDH in cardiac tissue. After I/R, higher levels of LDH in cardiac tissue were observed in groups of CSCs and CSC CM, implying less LDH released into coronary effluent and less severe myocardial damage in these hearts (Fig. 9A). However, using AMD3100 or SDF-1 siRNA resulted in lower levels of LDH remaining in the myocardium, indicating more severe cardiac damage. In addition, we observed that active caspase-3 levels were lower in CSC- or CSC CM- pretreated groups versus vehicle control after acute myocardial I/R, whereas blockade of SDF-1 receptor or reduction of SDF-1 release from CSCs increased cardiac caspase-3 activity (Fig. 9B), suggesting that SDF-1 is able to mediate acute protection in part through attenuation of cellular injury in response to myocardial I/R.

Following I/R injury, reactive oxygen species was produced in the heart. Hydrogen peroxide (H2O2), a reactive oxygen metabolic by-product, plays an important role in regulating oxidative stress-related states. Therefore, to elucidate the paracrine effect of CSCs on myocardial oxidative stress, we determined H2O2 production after I/R. We found that although there was a trend of decreased H2O2 production in CSCs or CSC CM-treated group, infusion of CSCs or CSC CM did not significantly reduce myocardial H2O2 production following acute I/R (Fig. 9C).

To determine whether CSC-secreted SDF-1 protects cardiomyocytes against injury, CSC CM was applied to cardiomyocytes (H9c2 cells, ATCC, Manassas, VA) subjected to hypoxia (1% O2) with or without AMD3100. After 24-hr of hypoxia, the supernatant was collected for an LDH assay and cell viability was determined by Trypan-blue exclusion. CSC CM protected cardiomyocytes from death following hypoxia as demonstrated by greater cell survival and lower levels of LDH in the supernatant compared to control (Fig. 9D). However, inhibition of SDF-1 receptor by AMD3100 neutralized this protection provided by CSC CM, indicating that CSC-derived SDF-1 is able to directly promote cardiomyocyte survival.

### Inhibition of myocardial STAT3 impaired CSC-mediated cardioprotection following I/R

**Figure 10:**
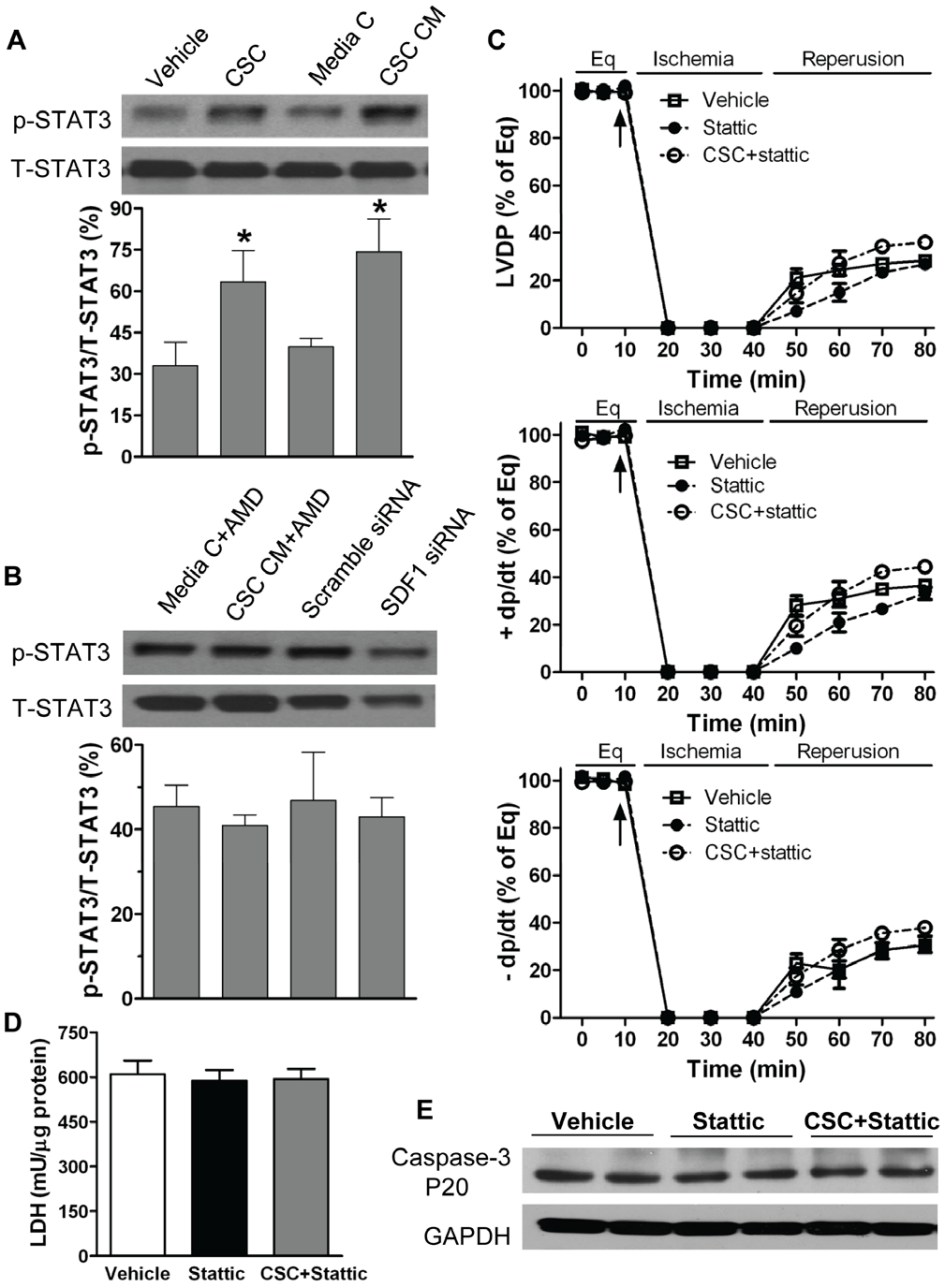
Myocardial STAT3 in CSC-mediated acute cardioprotection following I/R. A, Myocardial activation of STAT3 was determined in groups of Vehicle, CSCs, media control and CSC CM after I/R by Western blot assay. B, AMD3100 or SDF-1 siRNA neutralized CSC-induced STAT3 activation compared to their counterparts. Representative immunoblots of p-STAT3 and T-STAT3 were shown (one lane/group) and bar graph represents relative levels of p-STAT3/T-STAT3 ( %) in A and B. C, Stattic abolished CSC-mediated acute protection as demonstrate by unimproved LVDP and +/− dP/dt following I/R. D. Remaining LDH in myocardial tissue after I/R. E. Cardiac caspase-3 levels were determined in hearts treated with vehicle, stattic and CSC+stattic after I/R by Western blot. Shown are representative immunoblots (2 lanes/group). Results are Mean ± SEM, N = 4–5/group, *p<0.05 vs. vehicle or media control.

Studies have demonstrated that SDF-1-induced activation of Akt or STAT3 is involved in mediating cellular responses in a variety of cells [23], [26]–[28]. In addition, we have recently reported that SDF-1 conducts acute protection through myocardial STAT3 signaling in response to global I/R injury [24]. Here, to investigate by which pathway CSCs mediate acute protection in the heart subjected to I/R, we examined myocardial activation of STAT3. We observed that infusion of CSCs or CSC CM prior to ischemia significantly increased STAT3 activation following I/R (Fig. 10A). In addition, blockade of SDF-1 receptor or transfection of SDF-1 siRNA markedly decreased myocardial STAT3 activation (Fig. 10B). To further identify the role of STAT3 pathway in CSC-mediated acute protection in response to I/R, the STAT3 inhibitor-stattic was utilized. Inhibition of STAT3 significantly impaired post-ischemic myocardial function in CSC-treated group to a level seen in vehicle controls (Fig. 10C). Similarly, using stattic reversed CSC-reduced cellular damage in the heart subjected to I/R as exhibited by the level of cardiac LDH (Fig. 10D) and active caspase-3 expression (Fig. 10E).

### The role of Akt signaling in CSC-induced cardioprotecion in response to acute I/R

**Figure 11:**
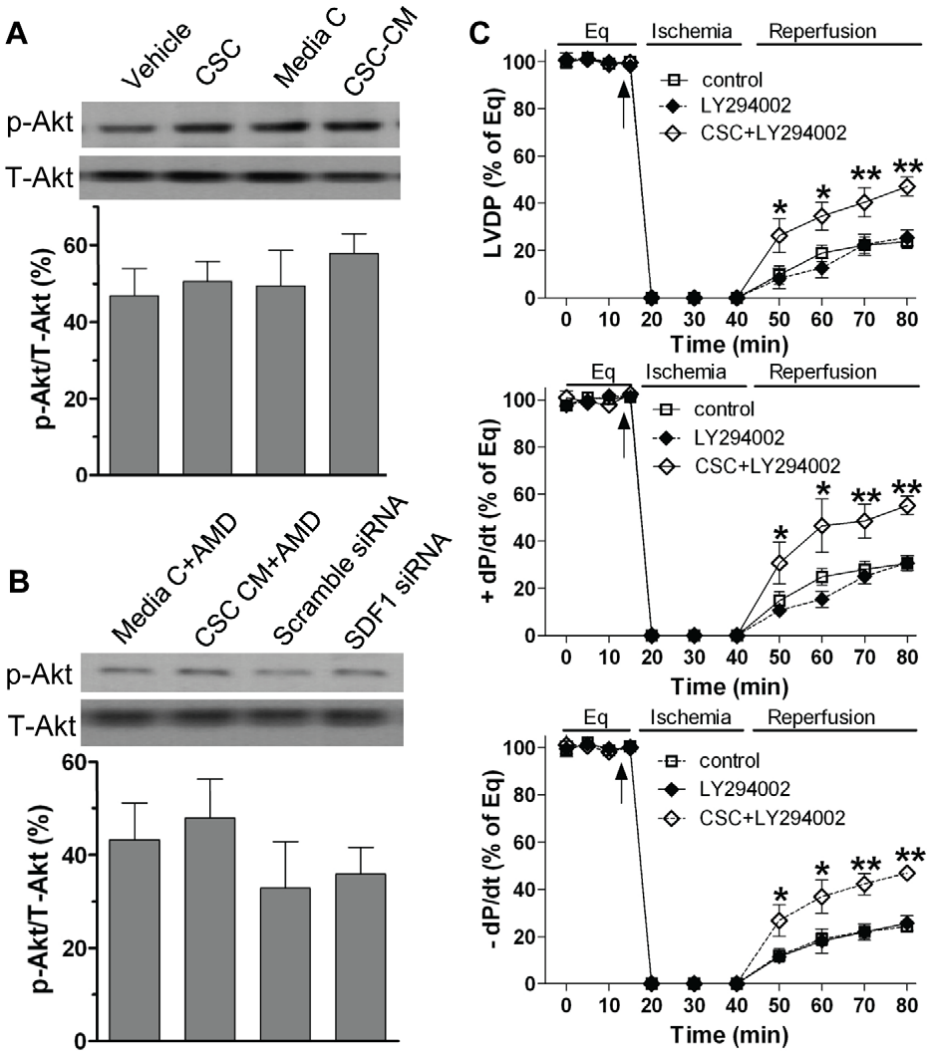
The Akt pathway in CSC-induced cardioprotection following I/R. A, Western blot assay revealed myocardial Akt activation in groups of Vehicle, CSCs, media control and CSC CM after I/R. B, The use of AMD3100 in CSC CM or SDF-1 siRNA in CSCs did not change myocardial Akt activation. Shown are representative immunoblots of p-Akt and T-Akt (one lane/group), and densitometry data of p-Akt represented as % of T-Akt. C, Inhibition of the Akt signaling by LY294002 did not affect CSC-improved post-ischemic LVDP, +dP/dt and –dP/dt. Results are mean ± SEM, n = 4-6/group, *p<0.05, **p<0.01 vs. control or LY294002 alone at the corresponding time point.

To investigate whether the Akt pathway plays a role in CSC-mediated acute protection in the heart subjected to I/R injury, we determined myocardial Akt activation. We observed that pretreatment with CSCs or CSC CM did not affect myocardial Akt activation (Fig. 11A). In addition, using SDF-1 receptor blocker in CSC CM or transfection of SDF-1 siRNA in CSCs did not change myocardial levels of phosphorylated-Akt (Fig. 11B). These results suggest that the Akt pathway is unlikely involved in mediating CSC-derived paracrine protection. To further rule out the contribution of Akt signaling in CSC-mediated acute protection, we utilized LY294002, an inhibitor of the Akt pathway. We observed that blockade of Akt signaling by using LY294002 did not attenuate CSC-improved myocardial function in response to acute I/R (Fig. 11C), further implying that CSC-induced paracrine protection is not mediated through the Akt pathway during I/R.

### Post-ischemic infusion of CSCs or CSC CM in protection of myocardial function

**Figure 12:**
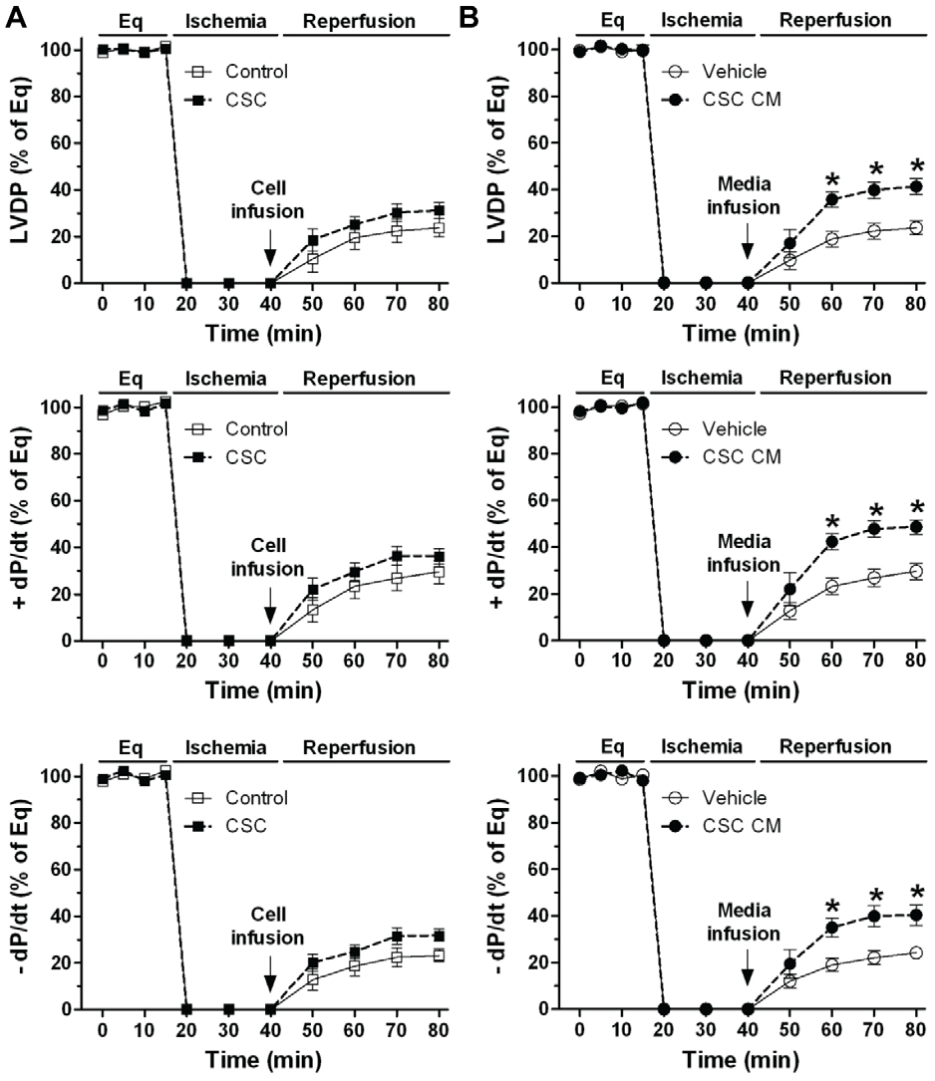
Post-ischemic infusion of CSCs or CSC CM in protection of myocardial function in response to I/R. LVDP and +/− dP/dt following I/R in groups of CSC and control (A), and groups of CSC CM and vehicle (B). Mean ± SEM, n = 5–6/group, *p<0.001 vs. vehicle at the corresponding time point. CM: conditioned medium.

To determine paracrine effect of CSCs on cardioprotection in a situation related to the clinical setting in which the ischemic damage is already present, we injected CSCs post-ischemically. However, we found that treatment of CSCs during the initiation of reperfusion resulted in many cells washed out of the myocardium by perfusion, and the cells stayed in the heart were not sufficient to produce adequate amounts of paracrine factors for cardioprotection. Our results indicated that although a trend of improved myocardial function was noticed in CSC-treated group following I/R, there was no significant difference (Fig 12A). To further identify the contribution of paracrine effect of CSCs on protection of myocardial function, we therefore infused CSC CM post-ischemically. We observed that post-ischemic infusion of CSC CM did significantly improve myocardial function as indicated in Figure 12B, suggesting that CSC-derived paracrine molecules are capable of inducing acute protection in a clinical-related setting.

## Discussion

In order to understand the paracrine action of CSCs in an ischemic enviroment, we conducted the present study which provided the initial evidence showing that CSC-derived SDF-1 mediated acute cardioprotection following I/R injury. Here, we reported that CSCs were capable of carrying on paracrine protection like MSCs did. SDF-1 was identified as one of the abundant paracrine factors in CSCs and played a critical role in CSC-mediated acute protection likely through STAT3 pathway following I/R.

The mammalian heart was originally thought to be a terminally differentiated organ without endogenous regenerative propeties after cardiac injury. However, accumulating studies have recently demonstrated that adult hearts contain a small number of cells expressing stem cell markers (Sca-1, c-Kit, MDR-1, etc.) [8], [12], [14], [17]. Such cells can differentiate into functional cardiomyocytes in vivo and in vitro. Transplantation of CSCs into infarcted myocardium has improved cardiac function and myocardial remodeling [8], [12], [19]. Therefore, the use of CSCs is promising for myocardial regeneration in the treatment of MI. However, the time period of at least several days is required for the CSC differentiation process. Before they develop into functional cardiomyocytes for repair purposes, CSCs may secret some molecules which are protective against myocardial injury. In fact, it is evident that paracrine factors play a major role in noncardiac stem cell-mediated cardioprotection after MI. However, the paracrine mechanism remains largely unclear after transplantation of CSCs into the ischemic heart. Herein, we observed that treatment of CSCs or CSC CM prior to ischemia significantly improved myocardial function very soon after I/R. In addition, in a setting related to the clinical situation, infusion of CSC CM during the initiation of reperfusion also protected myocardial function. These findings indicate that CSCs are capable of producing paracrine factors to mediate acute cardioprotection.

To date, it has been documented that many growth factors and cytokines derived from stem cells improve myocardial function, promote cell survival, advance ventricular remodeling and decrease tissue damage after myocardial ischemia. Among these, VEGF is a critical factor in angiogenesis and has been shown to facilitate stem cell paracrine protection in the ischemic myocardium [1], [29], [30]. Genetically modified bone marrow cells overexpressing VEGF are even more effective for cardioprotection [29]. In addition, our previous study has demonstrated that lowering VEGF levels alone by using VEGF siRNA in MSCs neutralizes MSC-induced protection of myocardial function following acute I/R [7]. Therefore, to elucidate the effect of CSC-derived VEGF on acute protection, we suppressed VEGF production in CSCs using VEGF siRNA. However, these CSCs were still able to improve myocardial function as scramble siRNA-transfected-CSCs did following I/R, indicating that VEGF is not critical to CSC-mediated acute protection.

We then asked the question whether CSCs differentiated to cardiomyocyte phenotype were still capable of conferring paracrine protection following I/R. We observed that pretreatment with DCSCs or DCSC CM did not protect the ischemic heart, suggesting that certain protective factors were derived from CSCs, but not DCSCs. Therefore, through direct comparison of the protein expression profiles of CSCs and DCSCs, SDF-1 was identified as one of the abundant paracrine factors in CSCs. In addition, CSCs secreted a larger amount of SDF-1 when compared to production of VEGF, HGF and IGF-1. In fact, previous research has already addressed that production of VEGF and HGF was not dominant in cardiac progenitor cells [19], [20]. Cardiosphere-derived cells have been shown to be incapable of producing HGF and IGF-1 [18].

Notably, when MSC-secreted VEGF dropped to the level of around 200 pg/ml by VEGF siRNA transfection, these MSCs did not provide protection of myocardial function following I/R injury [7]. In the current study, we have observed that CSCs and DCSCs produced VEGF at the level of <100 pg/ml and ∼200 pg/ml, respectively. This level of VEGF did not offer cardioprotection to DCSCs in response to I/R. In contrast, VEGF siRNA-transfected CSCs were still able to improve post-ischemic myocardial function. Taking them collectively, therefore, these findings suggest that a certain quantity of VEGF produced from stem cells is likely required to mediate protection. However, it still remains unclear whether the magnitude of cardioprotective effects is dependent on the quantity of other cytokines secreted from CSCs. In this regard, we determined whether abundantly secreted SDF-1 is critical to mediating CSC-induced acute cardioprotection. Herein, blockade of the SDF-1 receptor in the heart significantly attenuated CSC-induced paracrine protection following myocardial I/R. In addition, when SDF-1 expression in CSCs was downregulated by SDF-1 siRNA, pretreatment of CSCs did not improve post-ischemic cardiac function. Therefore, our data clearly indicate that SDF-1 is critical to CSC-mediated paracrine protection in hearts subjected to I/R.

Although the beneficial effects of SDF-1 have been mainly attributable to mobilization and recruitment of stem cells into an infarcted heart, recent studies have indicated that direct delivery of SDF-1 into the myocardium improved post-ischemic cardiac function within 24 hours of injury [26]. This favorable effect is likely due to preservation of myocardial tissue rather than through recruitment of stem cells [26]. In fact, SDF-1 has been shown to promote cell survival in a variety of cells including cardiomyocytes [23], [26]. In this study, our brief experiment period (a total of 65 minutes for I/R) limited the observation in the end-point of an influence of CSC-derived SDF-1 on programmed cell death after I/R. However, we did observe that CSCs or CSC CM decreased active caspase-3 levels and attenuated myocardial damage following I/R. In addition, blockade of the SDF-1 receptor in the heart or downregulated SDF-1 expression in CSCs abolished this protection in the myocardium. Furthermore, CSC CM protected cardiomyocytes from cell death following hypoxia, and inhibition of SDF-1 receptor neutralized this protection. These findings indicate that CSC-derived SDF-1 is able to mediate cardioprotection through alleviation of myocardial damage.

SDF-1 has been shown to upregulate Akt activation in cardiomyocytes and thus to promote cell survival after MI [23], [26]. Given that SDF-1 is a dominant paracrine factor in CSCs, it was postulated that CSC-induced paracrine protection may be mediated via increased Akt activation. However, in this study, infusion of CSCs or CSC CM did not upregulate cardiac Akt activation. In addition, neither blockade of SDF-1 receptor nor reduced SDF-1 levels in CSCs by transfection of specific SDF-1 siRNA downregulated myocardial activation of Akt after I/R. On the contrary, increased myocardial STAT3 activition was observed in CSC or CSC CM-treated hearts after I/R here. In addition, downregulation of SDF-1 pathway by inhibition of the SDF-1 receptor or transfection of SDF-1 siRNA in CSCs reduced cardiac activation of STAT3. In fact, it has been noted that CXCR4 contains two domains which are involved in regulating Jak2/STAT3 pathway [27]. Therefore, binding of SDF-1 to CXCR4 is able to trigger STAT3 activation, which then regulates cellular function in a variety of cells [27], [28]. Our recent study has also demonstrated that acute administration of SDF-1 prior to ischemia protects myocardial function through increased STAT3 activation following I/R injury [24]. In addition, SDF-1-increased STAT3 activation has been observed to improve cell growth and inhibit apoptosis [31], [32]. STAT3 is a protective factor in the heart and its activation mediates several down-stream signals, including upregulation of anti-apoptotic protein Bcl-2 or Bcl-xL [33]–[35], induction of heat shock proteins [36]–[38], production of angiogenic factors [39], and reduction of inflammatory cytokines [39]–[41]. Our findings here suggest that CSC-derived SDF-1 induces acute cardioprotection through upregulation of STAT3 following myocardial I/R. Furthermore, inhibition of STAT3 activity significantly impaired CSC-derived paracrine protection, implying the importance of STAT3 signal in mediating CSC-induced acute cardioprotection.

In this study, the Langendorff model was employed to characterize the paracrine effects of CSCs on myocardial function following global I/R. This isolated heart perfusion system obviates confounding effects of systemic actions including the effects of SDF-1on mobilization and recruitment of exogenous stem cells into the injured heart. In addition, this model has direct clinical relevance as global myocardial ischemia is employed during cardiac operations, such as cardiopulmonary bypass and cardioplegic arrest. However, further investigations are required using an in vivo MI model to completely understand the long-term effects of CSC-derived paracrine factors on angiogenesis, tissue regeneration and myocardial remodeling.

## Materials and Methods

### Ethics Statement

All animal studies conformed to the "Guide for the Care and Use of Laboratory Animals" (NIH publication No. 85–23, revised 1996). The protocols were reviewed and approved by the Indiana Animal Care and Use Committee of Indiana University.

### Preparation of cells and conditioned medium

Mouse CSCs were isolated from 6–7-week-old C57BL mice (Jackson Laboratories, Bar Harbor, ME) using a CSC isolation kit (Millipore, Billerica, MA) and were cultured in CSC maintenance medium (Millipore). After 2 weeks, the medium was changed to Iscove's Modified Dulbecco's Medium (GIBCO Invitrogen, Carlsbad, CA) with 10% fetal bovine serum and 1% pen-strep (IMDM completed medium) [9]. CSCs were measured for characteristics after one month. Differentiated CSCs (DCSCs) were obtained by culturing CSCs in cardiomyocyte differentiation medium (Millipore) for 9–10 days. Adult mouse cardiomyocytes were isolated from 8–10-week-old C57BL mouse hearts using a nonperfusion cardiomyocyte isolation system (Cellutron, Baltimore, MD). MSCs were isolated from 8–10-week-old C57BL mouse bone marrow and cultured as previously described [6], [42].

CSCs and DCSCs (1×10^6^) were plated in 75-cm flasks. After incubation for 6 hours in IMDM completed medium, cells were washed with PBS 2 times and cultured in 10 ml of serum-depleted IMDM. 24 hours later, supernatant was collected as conditioned medium (CM).

### Isolated mouse heart preparation (Langendorff Model)

A Langendorff perfusion system was utilized in isolated mouse hearts that were subjected to the same I/R protocol: 15-min equilibration followed by 25-min global ischemia (37°C) and 40-min reperfusion. Mouse hearts were isolated as our previously described [43]–[47]. Briefly, mice were anesthetized using isoflurane and heparinized (500 U i.p.), and hearts were rapidly excised via median sternotomy and placed in 4°C Krebs-Henseleit solution. The aorta was cannulated and the heart was perfused in the isovolumetric Langendorff mode (70 mmHg) and paced at 420 bpm/min except during ischemia. Data were continuously recorded using a PowerLab 8 preamplifier/digitizer (AD Instruments Inc., Milford, MA). The maximal positive and negative values of the first derivative of pressure (+ dP/dt and -dP/dt) were calculated using PowerLab software.

A total of 87 male C57BL mice (9–10-week-old) were randomly divided into (n = 5–6 hearts/group): 1) vehicle; 2) CSCs; 3) MSCs; 4) media control; 5) CSC CM; 6) scramble siRNA-transfected CSCs; 7) VEGF siRNA-transfected CSCs; 8) DCSCs; 9) DCSC CM; 10) Media control+AMD3100, a specific inhibitor of SDF-1 receptor (CXCR4), 5 µg/ml; 11) CSC CM+AMD 3100; 12) SDF-1 siRNA-transfected CSCs; 13) Stattic, an inhibitor of STAT3 activation, 20 µM; 14) CSCs+Stattic; 15) Media control+Ly294002, a specific inhibitor of Akt pathway, 2.5 µM; 16) CSC CM+Ly294002. Cells (0.1×10^6^ in 1 ml of perfusate), CM (1.5 ml) or vehicle was infused into the according isolated mouse hearts through a port above the aortic root within 1 minute immediately before ischemia. The drugs were infused into the isolated mouse hearts within 5 minutes before infusion of cells or CM. All doses of cells and drugs were chosen based on our preliminary studies and previous literature [6], [23], [24], [48]. In addition, concerning that the ischemic damage already occurs in the clinical setting, we further infused CSCs (0.1×10^6^ in 1 ml of perfusate) or CSC CM (1.5 ml) into ischemic hearts during the initiation of reperfusion to identify paracrine effect of CSCs on cardioprotection in a clinical point of view.

### Flow cytometric analysis

FITC-conjugated anti-CD45, PE-conjugated anti-Sca-1, CD29, CD31, CD44, CD45, and APC-conjugated CD117 (c-Kit) antibodies (Abs) were purchased from eBioscience (San Diego, CA) and BD Biosciences (San Jose, CA). The standard immunofluoresescent staining method was utilized and the percentage of cells expressing each cell surface antigen was analyzed by a FACSCalibur flow cytometer (BD Biosciences).

### RT Real-time PCR

Total RNA was extracted by using RNA STAT-60 (TEL-TEST, Friendswood, TX). 0.1 µg of total RNA was subjected to cDNA synthesis using cloned AMV first-strand cDNA synthesis kit (Invitrogen). Primer sequences are shown in Table 1. SYBR green real-time PCR amplification was performed on a 7300 real-time PCR system (Applied Biosystems, Foster City, CA).

**Table 1:** PCR primers.

Genes	Forward 5′-3′	Reverse 5′-3′
NKX2.5 AAAAGAGCTGTGCGCGCTGCAGAAGTAGACCTGCGCCTGCGAGAAGAGCAC Gata4CACCCCAATCTCGATATGTTTGATGACATTGCACAGGTAGTGTCCCGTCCCATC MEF2cCACGATGCCATCAGTGAATCAAAGGCTTGTCCTGGTAAAGTAGGAGTTGC Tbx5ACCAGAATCACAAGATCACACAGCTG CTCTTTACTTTGCATCCGAGACATCC α-MHCACCAGAGTTTGAGTGACAGAATGACGTGGGCCTCTAGGCGTTCCTTCTCTG MLC2vGAGAAACTTAAAGGGGCTGATCCTG AGCATCTCCCGGACATAGTCAGCCTTC cTnTAAACCCAAGCCCAGCAGGCTCTTCATGCTTCCTGTGGATGTCATCAAAGTCCAC Tnni3AAGAGCTTCAGGACTTATGCCGACAGCGTCAGATCTGCAATCTCAGTGATGTTC SDF-1TCTGCATCAGTGACGGTAAACCAG CTTGACGTTGGCTCTGGCGATGTGVEGF ACATCACCATGCAGATCATGCGGATCTCACATCTGCTGTGCTGTAGGAAGC HGFCTTTACGTTCACTTGCAAGGCCTTCCTCCACTTGACATACTATTGAAAGG IGF-1GCTTTTACTTCAACAAGCCCACAGGAAGCAACACTCATCCACAATGCCTG RPL7TTTGCCCTGAAGACACTTCGAAAGG TTCCTTGCCATCCTGGCCATCCGAATC β-actinAACCGTGAAAAGATGACCCAGATCATG ACAACACAGCCTGGATGGCTACGTAC		

### Western blot

Cells and heart tissues were lysed in cold RIPA buffer (Sigma, Saint Louis, MO). The protein extracts (10–30 µg) were subjected to electrophoresis on a 4–12% precise protein gel (Invitrogen) and transferred to a nitrocellulose membrane. The membranes were incubated with the following primary Abs: Gata4 (Santa Cruz Biotechnology Inc., Santa Cruz, CA), Sarcomeric (SM) α-actin (Sigma), Akt, phosphor-Akt (p-Akt), STAT3, phosphor-STAT3 (p-STAT3) (Cell Signaling Technology, Beverly, MA), caspase-3 (Santa Cruz Biotechnology, Inc., Santa Cruz, CA) and GAPDH (Biodesign International, Saco, Maine), followed by horseradish peroxidase-conjugated goat anti-rabbit or anti-mouse secondary Ab and detection using SuperSignal West Pico stable peroxide solution (Pierce, Rockford, IL). Films were scanned using an Epson Perfection 3200 Scanner (Epson America, Long Beach, CA).

### Immunofluorescent staining

The cells grown on the chamber slide system were fixed and stained with anti-Gata4 and anti-SM α-actin Abs using a standard immunohistochemistry protocol followed by incubation with Texas red-conjugated anti-rabbit or Fluorescein-conjugated anti-mouse Abs (Vector Laboratory, Burlingame, CA). DAPI was used for nuclear staining. Cell morphology and fluorescence were examined under an inverted fluorescence microscope (Nikon TE2000U, Nikon, Melville, NY). Images were digitalized with QCapture (QImaging, Surrey, BC).

### Cytokine Ab array

Cytokine secretion was determined in conditioned medium from CSCs and DCSCs by using a cytokine Ab array according to the manufacturer's instructions (R&D Systems Inc., Minneapolis, MN). The signals were detected by using SuperSignal West Pico stable peroxide solution (Pierce). Signal densities were compared using TotalLab software (Nonlinear USA, Inc., Durham NC).

### siRNA transfection

VEGF siRNA [7], SDF-1 siRNA and scrambled-control siRNA were purchased from Dharmacon (Lafayette, CO). Lipofectamine 2000 (Invitrogen) was used to transfect these siRNAs into CSCs based on a standard transfection method. CSCs were plated in 12-well plate at 0.5×10^5^/well/ml on day 1. On day 2, cells were transfected with according siRNAs using standard procedure. After 1 day of transfection (day 3 in culture), complexes containing siRNA were washed out, normal IMDM medium was added, and the cells were allowed to incubate for an additional 2 days. On day 5, the supernatant was collected for the measurement of VEGF and SDF-1 by using ELISA, and CSCs were harvested for Langendorff experiments.

### ELISA

The supernatants collected from cell culture were measured for production of SDF-1, VEGF, HGF and IGF-1 using according ELISA kit (R&D). In addition, protein levels of SDF-1 were also determined between CSCs and cardiomyocytes that were isolated from adult mouse hearts and cultured for 2 hours in vitro.

### Measurement of cell/tissue damage

Cell viability was analyzed by Trypan-blue exclusion and the fraction of blue cells was quantified by light microscopy. LDH activity was assessed to determine the extent of cellular injury using a Cytotoxicity Detection Kit (Roche Diagnostics Corporation, Indianapolis, IN).

### Myocardial oxidative stress

Heart samples were prepared as described above in the section of Western blot. Oxidative stress after I/R was determined by myocardial H2O2 production using a Red Hydrogen Peroxide Assay kit (Enzo Life Sciences International, INC., Plymouth Meeting, PA) according to the manufacturer's instructions.

### Presentation of data and statistical analysis

All reported values are mean ± SEM. Data were compared using two-way analysis of variance (ANOVA) with post-hoc Bonferroni test or Student's t-test. A probability value of less than 0.05 was considered statistically significant.
